# The Clinical and Nonclinical Values of Nonexercise Estimation of Cardiovascular Endurance in Young Asymptomatic Individuals

**DOI:** 10.1100/2012/958752

**Published:** 2012-04-19

**Authors:** Mahmoud A. Alomari, Dana M. Shqair, Omar F. Khabour, Khaldoon Alawneh, Mahmoud I. Nazzal, Esraa F. Keewan

**Affiliations:** ^1^Division of Physical Therapy, Department of Rehabilitation Sciences, Jordan University of Science and Technology, Irbid 22110, Jordan; ^2^Department of Nutrition and Food Technology, Jordan University of Science and Technology, Irbid 22110, Jordan; ^3^Department of Medical Laboratory Sciences, Jordan University of Science and Technology, Irbid 22110, Jordan; ^4^Department of Internal Medicine, Faculty of Medicine, Jordan University of Science and Technology, Irbid 22110, Jordan; ^5^Division of Rheumatology, Department of Medicine, King Abdulla Hospital, Irbid 22110, Jordan; ^6^Department of Rehabilitation Medicine, Faculty of Medicine, Jordan University of Science and Technology, Irbid 22110, Jordan; ^7^Department of Rehabilitation Medicine, King Abdulla Hospital, Irbid 22110, Jordan; ^8^Department of Physiology, Faculty of Medicine, Jordan University of Science and Technology, Irbid 22110, Jordan

## Abstract

Exercise testing is associated with barriers prevent using cardiovascular (CV) endurance (CVE) measure frequently. A recent nonexercise model (NM) is alleged to estimate CVE without exercise. This study examined CVE relationships, using the NM model, with measures of obesity, physical fitness (PF), blood glucose and lipid, and circulation in 188 asymptomatic young (18–40 years) adults. Estimated CVE correlated favorably with measures of PF (*r* = 0.4 − 0.5) including handgrip strength, distance in 6 munities walking test, and shoulder press, and leg extension strengths, obesity (*r* = 0.2 − 0.7) including % body fat, body water content, fat mass, muscle mass, BMI, waist and hip circumferences and waist/hip ratio, and circulation (*r* = 0.2 − 0.3) including blood pressures, blood flow, vascular resistance, and blood (*r* = 0.2 − 0.5) profile including glucose, total cholesterol, LDL-C, HDL-C, and triglycerides. Additionally, differences (*P* < 0.05) in examined measures were found between the high, average, and low estimated CVE groups. Obviously the majority of these measures are CV disease risk factors and metabolic syndrome components. These results enhance the NM scientific value, and thus, can be further used in clinical and nonclinical settings.

## 1. Introduction

Diseases of the cardiovascular (CV) system are the number 1 cause of mortality and morbidity in the world and will continue to be for years to come [1]. Reduced CV endurance (CVE) increases the risk of hypertension, obesity, diabetes, and coronary, cerebral, and peripheral vascular diseases in a variety of populations [2, 3]. Recently, 12.2% of myocardial [4] and 28.5% of cerebral [5] infarctions were attributed to lack of physical activities in 2 large-scale studies involving 74 countries. Additionally, studies have found that determining CVE can predict all-cause and CV-related mortality and morbidity in children and young and older adults with and without CV diseases (CVD). Therefore, many have recommended to include CVE testing in the screening for CVDs [6, 7]. However, the risk, time constrains, inconvenience, and cost associated with exercise testing prevent from using the maximal oxygen consumption (VO_2_ max) measure, the direct and golden standard for CVE, frequently [8, 9].

The previously developed [10] and recently cross-[11] and criterion- [12] validated nonexercise model (NM) has been found reasonably accurate to estimate CVE [13]. The model is based on the regression of independent and objective variables recognized to be related to CVE and CVD, such as gender, age, body mass index (BMI), resting heart rate (HR), and self-reported habitual physical activity (PA) levels. The method is proposed to be a valid and simple measure of CVE that can be used in clinical setting without the difficulties of exercise testing [13]. However the relationships of NMVO_2_ max with CVD risk factors and metabolic syndrome components have not been examined.

Therefore, the study evaluated the relationships of NMVO_2_ max with measures of physical fitness (PF), obesity, circulatory, and blood glucose and lipid profile in young asymptomatic individuals. Given previous findings [2, 7, 13, 14], these measures are expected to be related to NMVO_2_ max. Indeed, the majority of these measures are related to CVD and the metabolic syndrome. These relationships are particularly important for future clinical and nonclinical considerations of the model. Especially in the screening for CVDs asymptomatic individuals can benefit from primary prevention programs to avoid or at least delay CVDs [15].

## 2. Methodology

### 2.1. Participants and Study Design

Asymptomatic apparently healthy males and females between 18 and 40 year old were invited to participate in this cross-sectional study. The exclusion criteria were fasting plasma triglycerides ≥ 150 mg /dL; HDL-C < 40/50 mg/dL (men/women); glucose ≥ 110 mg/dL; or BP ≥ 130/85 mmHg [16]. Additionally, smokers and individuals with metabolic and CVDs or taking medications that might affect vascular function, BP, or blood glucose and lipid profile were excluded from the study. After a detailed orientation of the experimental procedures, possible risks, benefits, and requirements, all individuals signed an informed consent approved by the institutional review board.

### 2.2. Health-Related Fitness Measures

#### 2.2.1. Anthropometrics

Body weight (Bweight) in kilograms/the squared height in meters was used to calculate BMI [17]. Additionally, the participants' percent body fat (Fpercent) and body water (Bwater) were measured using bioelectrical impedance (Microlife WS 100, Microlife AG, Heerbrugg, Switzerland). Subsequently fat mass (Fmass) was calculated as Fpercent ∗ Bweight whereas muscle mass (Mmass) was calculated as the difference between Bweight and Fmass [17]. The ratio of the waist and hip (WHR) was calculated after determining the waist (WC) and hip (HC) circumferences at the umbilicus height and at the greatest circumference in the pelvic bone, respectively [17].

#### 2.2.2. Physical Fitness

Handgrip (HGS), shoulders press (Shpress) and leg extension (Lgext) strengths as well as the 6-minute walk test (6 MWD) were used as measures of physical fitness. The average HGS was calculated after 3 all-out griping trials in the dominant hand using a dynamometer [17]. Each participant walked as fast as possible to examine the maximum distance achieved in 6 minutes (6 MWD) [18]. Shoulder press (Shpress) and leg extension (Lgext) machines were used to determine upper and lower body strengths, respectively. The weight-lifted 2–20 repetitions were applied to the “one repetition maximum (1-RM)” equation to determine maximal strength. The 1-RM equation = kg at #RM 2–20/[1.00–(#RM∗0.02)], given that #RM = number of repetitions performed, 1.00 = 100% as a decimal, and 0.02 = 2% as a decimal. The 1-RM was then multiplied by 70% to determine Shpress, and Lgext endurances [17].

### 2.3. Circulatory Measures

#### 2.3.1. Blood Pressure

Automated noninvasive auscultatory (Omron HEM-907XL, Omron Healthcare, Inc, Bannockburn, Illinois) BP measurements for the systolic (SBP) and diastolic (DBP) pressures and HR were obtained from the participants after 20 minutes of supine resting. Pulse pressure (PP) was calculated as SBP-DBP whereas mean arterial pressure (MAP) was calculated as DBP + (1/3∗PP) [17].

#### 2.3.2. Vascular Function

Vascular function measurements were explained previously in details [19]; however they will be briefly described. The participants' blood flows (BF) at rest (RBF) and after reactive hyperemia (RHBF) were measured in the dominant forearm with strain-gauge plethysmography, from a supine position after 20 minutes resting. Forearm RBF was measured immediately after releasing a pressure of 7 mmHg below DBP applied with another tourniquet placed above the elbow. The RHBF was obtained after occluding the upper arm arterial circulation for 5 minutes. The calculations, for RBF and RHBF, were made from the slop drawn at the 1st 2-3 beats of the volume graph. Subsequently, vascular resistance (Vr) at rest (RVr) and after 5 minutes of upper arm arterial occlusion (RHVr) was calculated as MAP/Vr [19].

### 2.4. Glucose and Lipid Profile Measures

Blood samples after overnight fasting were drawn from each participant by venipuncture into 5 mL plain tubes for serum lipids and triglycerides determination and into 4 mL EDTA containing tubes for blood glucose and lipid profile (cholesterol, HDL-C, LDL-C, HDL/LDL, triglyceride) measurements. The serum was separated from blood cells by centrifugation at 2000 ×g for 10 min. All biochemical parameters were assayed in the laboratory of King Abdulla University Hospital, Irbid, Jordan using Roche Chemistry Analyzer and reagents (Roche Diagnostics, Indianapolis, IN, USA). For all assays, the intra-assay coefficient of variation was less than 2.6% [20].

### 2.5. Physical Activity Level

The short-form Arabic version of the international PA questionnaire (IPAQ) was used to obtain the level of PA [21]. The subjects were classified into low, medium, and high PA levels, according to total PA participation. The classifications were according to <600, 601–1500 and >1501, and assigned the values 1, 2, and 3, respectively [21]. These values were then used in the non exercise regression equation to estimate VO_2_ max [10].

### 2.6. Estimation of VO_2_ Max Using the Nonexercise Regression Model

The regression model, originally developed by Jackson, was used to estimate CVE according to VO_2_ max = [Gender(female = 0; male = 1) ∗ 2.77] − [Age ∗ 0.10] − [BMI ∗ 0.17] − [resting HR ∗ 0.03] + [PA level ∗ 1] + 18.07. Subsequently, the participants were classified into poor, average, and high level of CVE [10].

### 2.7. Statistical Analysis

Pearson's correlations were used to examine the relationships of NMVO_2_ max with PF, circulatory, and blood glucose and lipid profile measures. Additionally, after dividing the participants into poor, average, and high CVE groups, 1-way ANOVA tests were used to examine the difference between the 3 groups in these measures.

## 3. Results

### 3.1. Participants

As in Table 1, 188 Arab individuals participated in the study including 90 males and 98 females with estimated VO_2_ max of 51.15 ± 4.6 and 42.0 ± 5.0 mL·kg^−1^·min^−1^, respectively. The distribution of poor, average, and high estimated VO_2_ max groups was 6.4 (*n* = 12), 55.8 (*n* = 105), and 37.7% (*n* = 71), respectively, with estimated VO_2_ max 34.1 ± 7.1, 43.9 ± 4.1, and 53.1 ± 4.5 mL·kg^−1^·min^−1^, respectively. The number of males and females in the poor, average, and high estimated VO_2_ max groups was 1, 36, and 53, and 11, 69, and 18, respectively.

### 3.2. Estimated VO_2_ Max Relationships with Health-Related Fitness Measures

Tables 2 and 3 show that estimated VO_2_ max correlated with HGS, 6 MWD, and Shpress and Lgext strength and endurance, as well as with Bweight, Fpercent, Fmass, Bwater, and WHR (*r* value range: 0.2–0.7). Differences (*P* value range: 0.05–0.000) between the 3 groups in HGS, 6 MWD, Spress and Lgext are shown in Table 4. Similarly, Table 4 demonstrates differences (*P* value range: 0.02–0.000) between the 3 groups in Bweight, BMI, Fpercent, Fmass, Mmass, Bwater, WC, and HC. 

### 3.3. Estimated VO_2_ Max Relationships with Circulatory Measures 

**Figure 1:**
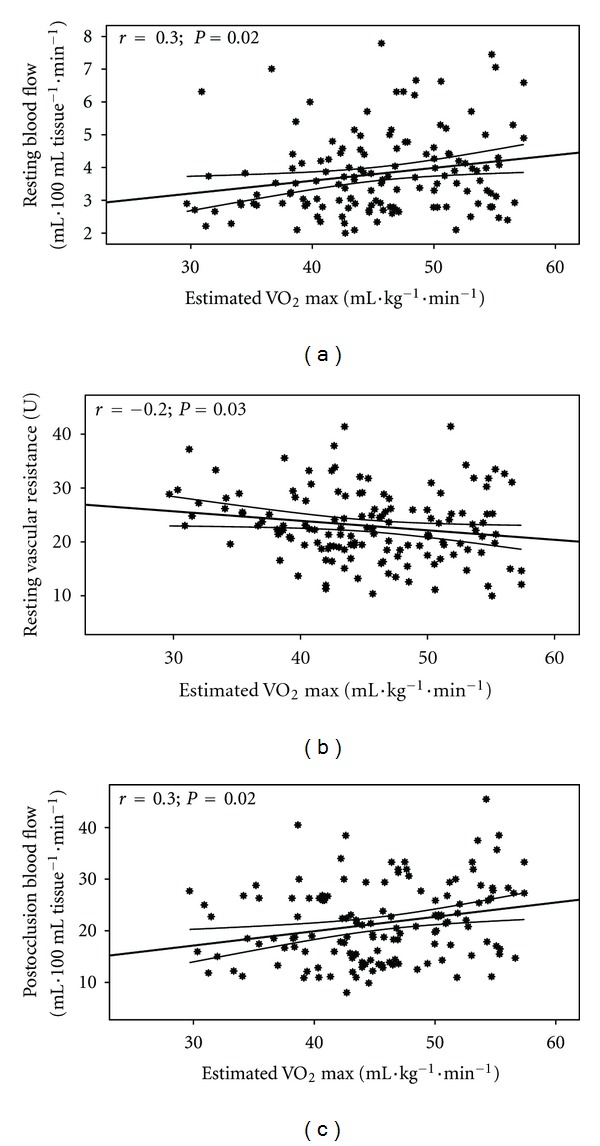
Estimated VO_2_ relationship with resting blood flow (a) (*r* = 0.3; *P* = 0.02), resting vascular resistance (b) (*r* = −0.2; *P* = 0.03), and postocclusion blood flow (c) (*r* = 0.3; *P* = 0.02).

**Figure 2:**
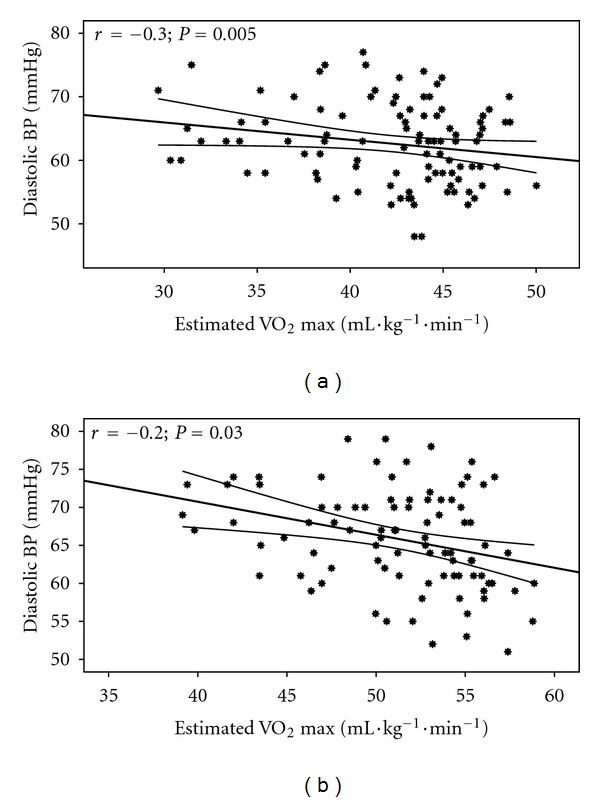
Estimated VO_2_ relationship with DBP in the females (a) (*r* = −0.3; *P* = 0.005) and males (b) (*r* = −0.2; *P* = 0.03).

**Figure 3:**
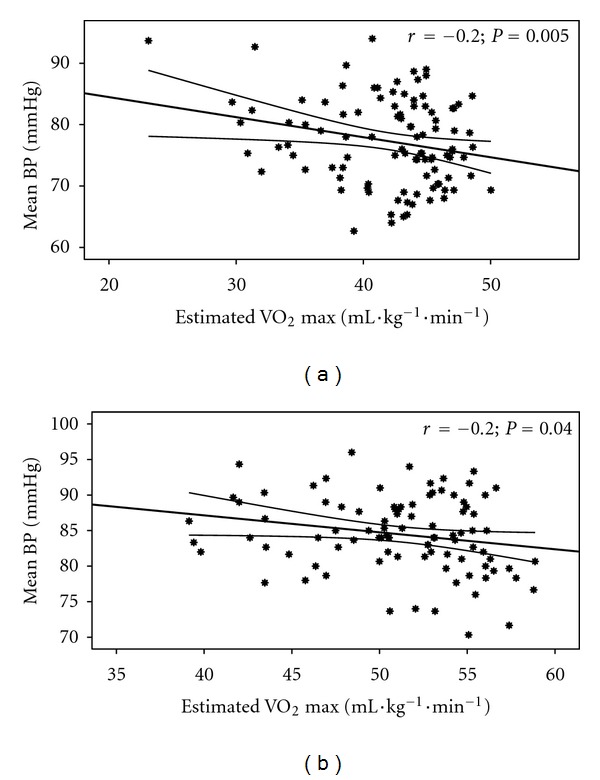
Estimated VO_2_ relationship with MAP in the females (a) (*r* = −0.2; *P* = 0.005) and males (b) (*r* = −0.2; *P* = 0.04).

**Figure 4:**
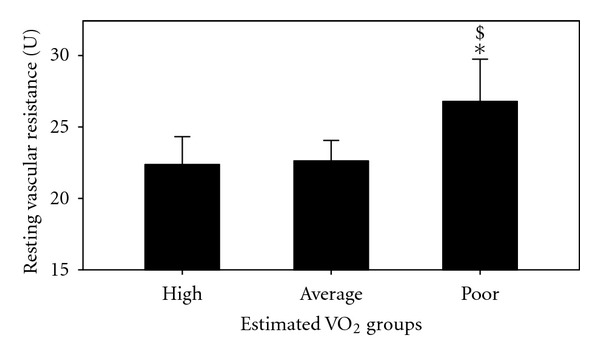
Resting vascular resistance in the poor, average, and high estimated VO_2_ max groups. *: *P* < 0.05 versus average and ^$^: *P* < 0.05 versus high groups.

**Figure 5:**
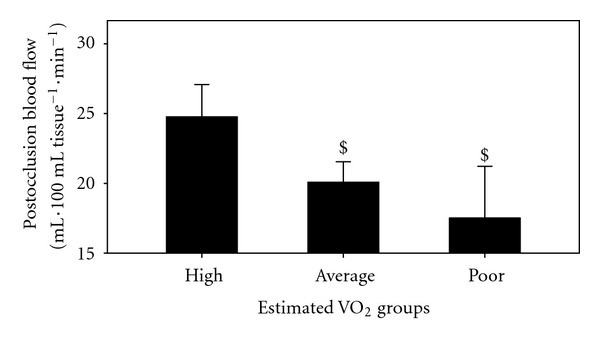
Postocclusion blood flow in the poor, average, and high estimated VO_2_ max groups. ^$^= *P* < 0.05 versus high groups.

Estimated CVE relationships with BF and Vr are shown in Figure 1 and with DBP and MAP are shown in Figures 2 and 3. Additionally, differences between the 3 groups in RVr (*P* = 0.05) and RHBF (*P* = 0.000) are shown in Figures 4 and 5. 

### 3.4. Estimated VO_2_ Max Relationships with Blood Glucose and Lipid Profile

**Figure 6:**
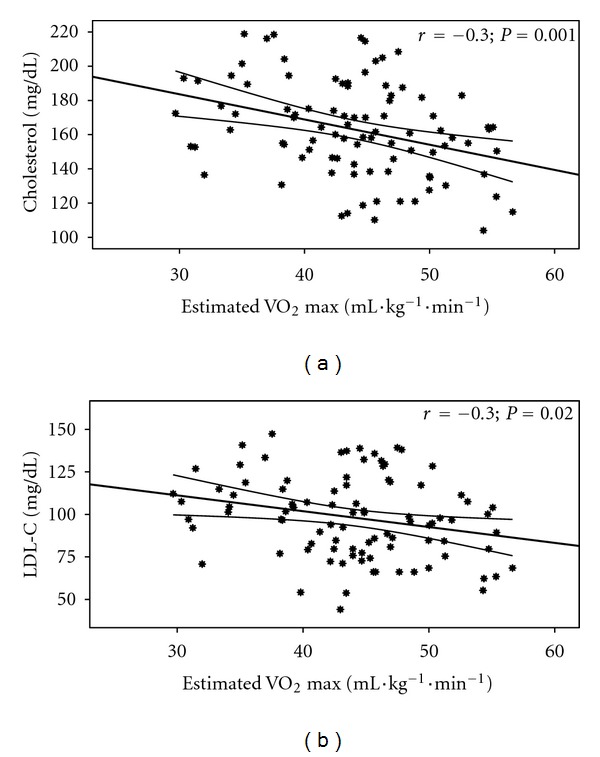
Estimated VO_2_ relationship with cholesterol (a) (*r* = −0.3; *P* = 0.001) and LDL-C (b) (*r* = −0.3; *P* = 0.02).

**Figure 7:**
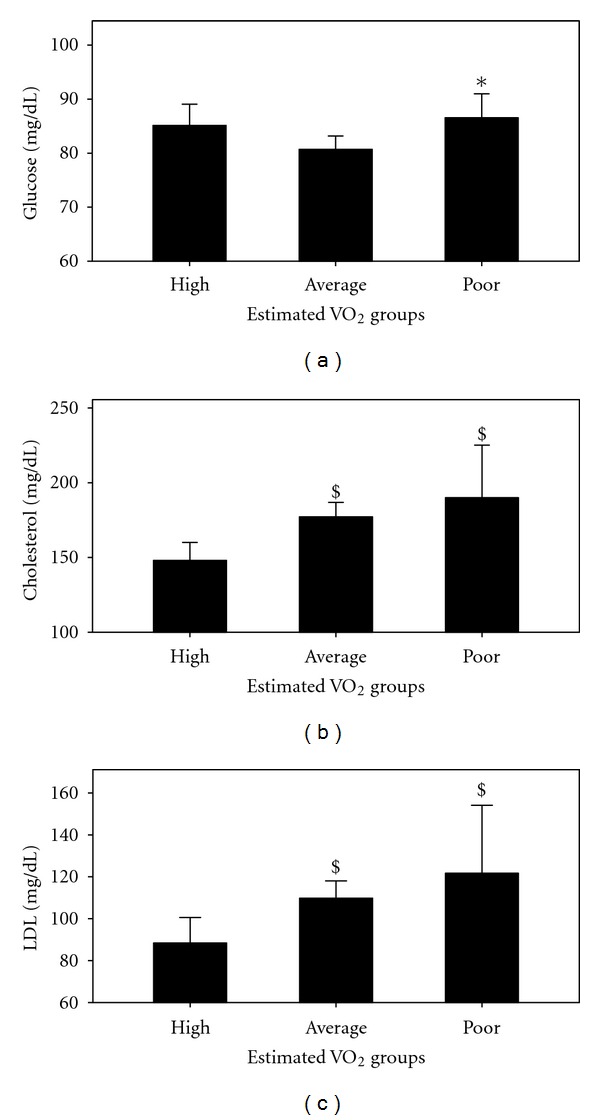
Blood glucose (a), cholesterol (b), and LDL-C (c) in the poor, average, and high estimated VO_2_ max groups. *= *P* < 0.05 versus average group; ^$^= *P* < 0.05 versus high group.

Estimated VO_2_ correlated favorably with blood glucose, cholesterol, HDL-C, LDL-C, HDL/LDL, and triglyceride in the males and females (Table 5) separately and with cholesterol (Figure 6(a)) and LDL-C (Figure 6(b)) for the males and females combined.  Figure 7 revealed that blood glucose, cholesterol, and LDL-C were different (*P* < 0.05) between the 3 groups.  Table 6 shows that blood cholesterol, HDL-C, LDL-C, HDL/LDL, and triglyceride among the males were different (*P* < 0.05) between the high and average groups. 

## 4. Discussion

Estimating VO_2_ max using the regression model was related to PF, body composition, circulatory, and blood glucose and lipid profile measures. Additionally, participants in the higher estimated VO_2_ max category were at lower risk profile. Obviously, the majority of these variables are established CVD risk factors and components of the metabolic syndrome. Therefore, these results further enhance the value of the regression model and thus can be used to estimate CVE in large-population settings. 

Extensive efforts have recently been put forward to identify asymptomatic persons with or without CV risk factors to qualify for primary prevention using various diagnostic tools [8, 22–25]. The diagnostic value of graded exercise testing (GXT) was recognized some time ago to record changes in physiological vitals (i.e., HR, BP, and respiratory rate) for patients with CVDs during maximal exertion [26, 27]. Finding these relationships is quite remarkable, especially that the participants were young and without CVDs or even risk factors. These results indicate that NMVO_2_ max seems to be useful to differentiate between individuals according to CVD risk factor profile and status of the metabolic syndrome. This is particularly important considering that previous ample evidences have suggested the possibility of using CVE testing in risk stratification for primary prevention in asymptomatic individuals [6, 7]. The results of these studies suggest an independent prognostic and diagnostic values of maximal exercise capacity (i.e., CVE) regardless of age, gender, race, and health status [28]. 

In one of the earliest large-scale studies (>50,000 individuals) greater CVE was associated with 43 and 53% lower all-cause mortality, as well as 47 and 70% lower CV-related mortality in men and women, respectively [29, 30]. More recently, a meta-analysis for >102,000 individuals concluded a 13 and 15% lower all-cause and CV-related mortalities, respectively, with every 1 MET increase in CVE level. The authors further elaborated that a 1 MET increase in CVE level was comparable to a decrement 7 cm in waist circumference, 5 mmHg in SBP, 1 mmol/L in triglyceride, 1 mmol/L in plasma glucose, and a 0.2 mmol/L increment in HDL-C [31]. 

Assessment of CVE is also valuable and widely used in quite few nonclinical settings for a variety of purposes. It can be utilized to predict future performance, is baseline for exercise prescription, is to evaluate the efficiency of exercise programs, and is helpful for positive motivation [32]. The relationships of estimated VO_2_ max with muscle mass, upper and lower body strength, HGS, and 6 MWD measures, found herein, suggest that NMVO_2_ max can also be an acceptable indicator for PF confirming previous findings [11–13, 33]. This is vital and indicates that NMVO_2_ max might be a simple alternative tool to estimate CVE in young asymptomatic individuals for nonclinical purposes. 

Forearm BF and Vr in the current study are associated with greater estimated VO_2_ max in young asymptomatic individuals confirming the importance of the arterial system for muscle BF to achieve greater VO_2_ and energy during maximum performance. Essentially, improved blood delivery to the working muscle results in reduced workload placed on all components of the CV system at any given intensity, including resting. This decrease in workload allows the CV system to function efficiently for longer period of time, delays fatigue, increases exercise tolerance, and is essential for healthy CV system [34, 35]. 

However, despite these compelling evidence confirming the clinical and nonclinical values of determining CVE [28–32, 36–39], it is largely underutilized, especially for CVD risk stratification. Rightfully so, one might argue that lengthy, inconvenient, and risky producers of maximal exertion during exercise testing might be the reason for not using CVE regularly. Alternatively, the current regression model is claimed to estimate CVE without the “burdens” associated with “conventional” exercise testing. The research group, who originally developed the equation, asserts that the model is relatively simple, low-cost, and low-risk estimate of CVE and could be used in clinical, nonclinical, and research settings. Additionally, the estimation of VO_2_ max with the model can serve as a baseline for designing exercise prescriptions, monitoring adaptations to exercise, and measuring the success of exercise programming [10, 11, 13]. One slight deviation from the original model is estimating CVE in the current study with the IPAQ, which classifies the participants into 3 versus 5 levels used in the original equation [10, 13]. We think this modification can make the model more versatile and acceptable in wider-range settings. 

Needless to say, repeatedly challenging the body with exercise results in adaptations of various body systems to assure proper energy production during subsequent physical exertion. Habitual exercise training is associated with increased CV endurance, HGS, and upper and lower body strengths. Similarly, overwhelming data demonstrate that regular participation in exercise training results in changes in body composition measures. Favorable alterations in weight, abdominal obesity, percent body fat, BMI, and fat-free mass have been observed even after low-intensity exercise (i.e., walking). Though the mechanism(s) for these adaptations are not entirely known, exercise seems to increase metabolic rate thereby energy expenditure, even during resting [40–44]. Changes in blood glucose and lipid profile are also well established following exercise training, especially aerobic. Blood glucose, cholesterol, LDL-C, and triglyceride are lower whereas HDL-C is greater in physically active individuals [40–44]. Regular exercise also improves muscle blood flow and VO_2_ to meet the metabolic demands in subsequent exercise sessions. The improved blood flow has been attributed to endothelium-dependent and -independent structural and functional changes. These changes include enhanced endothelial [45] and metabolically [46] mediated vasodilatations, arterial smooth muscle responsiveness [47], and reduced norepinephrine [48] and endothlin1-mediated arterial constrictions [49, 50]. Nonetheless, since the CV system is a continuum, the improvements in the capabilities of various components directly interact with each other to enhance O_2_ delivery to the working muscles [35, 51, 52]. Alterations in blood and stroke volumes, cardiac dimensions and output, and neurohormonal balance have also been observed after aerobic exercise [53, 54]. 

## 5. Conclusion

Estimation of cardiovascular endurance was related to CVD risk factors and components of the metabolic syndrome including measures of obesity, PF, circulatory, and blood glucose and lipid profile. Thus, the data indicate that the NM, evaluated herein, can be potentially used as an indirect measure of VO_2_ max to screen for CVD risk factors. These results further enhance the scientific value of the NM to be used more in clinical and nonclinical settings. Finally, the findings confirm that CVE is useful for weight control, physical capacity, CV risk factor management and general CV health.

## Figures and Tables

**Table 1:** Participant characteristics (*n* = 188).

Variable	Mean ± SD	Range
Age (years)	24.04 ± 5.3	18–40
Weight (kg)	69.7 ± 15.8	41–119
Height (cm)	168.6 ± 9.5	146–189
Body mass index	24.35 ± 4.3	16.5–37.3
Estimated VO_2_ (mL·kg^−1^·min^−1^)	46.4 ± 6.7	23.1–58.9

**Table 2:** Estimated maximum VO_2_ relationship with physical fitness measures.

	Physical fitness measures	*r* value	*P* value
Estimated VO_2_ max	Handgrip strength	0.5	0.000
	6 MWD	0.5	0.000
	Leg extension 1RM	0.4	0.000
	Shoulder press 1RM	0.5	0.000

**Table 3:** Estimated maximum VO_2_ relationship with body composition measures in the males and females.

	Body composition measures	Gender	*r* value	*P* value
	Weight	Males Females	−0.5 −0.6	0.000 0.000
	Body fat percent	Males Females	−0.6 −0.6	0.000 0.000
	Body fat mass	Males Females	−0.6 −0.7	0.000 0.000
Estimated VO_2_ max	Body water content	Males Females	0.3 0.4	0.020 0.006
	Waist circumference	Males Females	−0.5 −0.5	0.000 0.000
	Hip circumference	Males Females	−0.2 −0.6	0.030 0.000
	Waist/hip	Males Females	−0.5 −0.2	0.000 0.040

**Table 4:** Physical capacity and body composition measures in the high, average, and low estimated VO_2_ groups.

Measures	Estimated VO_2_ groups			ANOVA
	High (*n* = 71)	Average (*n* = 105)	Low (*n* = 12)	
Handgrip strength (kg)	31.9 ± 11.1	29.3 ± 13.2	24.6 ± 11.1^∗$^	0.020
Maximal walked distance (m)	654.9 ± 92.8	579.0 ± 82.2^$^	517.3 ± 65.4^∗$^	0.000
Leg extension 1-RM (kg)	41.00 ± 18.1	36.10 ± 21.0^$^	31.40 ± 10.6^$^	0.050
Shoulder press 1-RM (kg)	41.60 ± 15.3	34.80 ± 17.0^$^	30.80 ± 9.2^$^	0.040
Weight (kg)	67.4 ± 13.0	69.9 ± 16.6	82.2 ± 17.6^∗$^	0.020
Body mass index	22.2 ± 3.2	24.5 ± 4.2^$^	30.0 ± 4.6^∗$^	0.000
Body fat percent (%)	16.7 ± 5.8	25.2 ± 6.1^$^	32.3 ± 5.8^∗$^	0.000
Body fat mass (kg)	11.4 ± 4.8	18.0 ± 7.0^$^	27.0 ± 8.5^∗$^	0.000
Body muscle mass (kg)	56.2 ± 10.7	52.1 ± 12.0^$^	55.3 ± 11.4	0.040
Water content (kg)	62.0 ± 5.6	54.0 ± 3.4^$^	54.7 ± 3.5^$^	0.000
Waist circumference (cm)	78.8 ± 10.0	80.8 ± 13.3	90.7 ± 15.1^∗$^	0.009
Hip circumference (cm)	99.2 ± 7.5	102±10.2^$^	111.2 ± 9.8^∗$^	0.000
Waist/hip	0.8 ± 0.07	0.8 ± 0.09	0.8 ± 0.1	0.620

Values are in mean ± SD. *: *P* < 0.05 versus the average; ^$^: *P* < 0.05 versus the high group.

**Table 5:** Estimated maximum VO_2_ relationship with blood glucose and lipid profile in the males and females.

		Glucose	Cholesterol	HDL-C	LDL-C	HDL/LDL	Triglyceride
Estimated VO_2_	Males	−0.04; 0.8	−0.4; 0.01*	0.3; 0.03*	−0.3; 0.02*	0.3; 0.03*	−0.5; 0.002*
	Females	−0.4; 0.007*	−0.3; 0.03*	0.1; 0.5	−0.3; 0.03*	0.3; 0.05*	−0.3; 0.03*

*: significantly correlating with estimated maximal oxygen consumption.

**Table 6:** Blood glucose and lipid profile for the males in the high and average estimated VO_2_ groups.

	High (*n* = 53)	Average (*n* = 36)	*P* value
Glucose (mg/dL)	87.7 ± 6.8	81.2 ± 11.2	0.200
Cholesterol (mg/dL)	173.3 ± 20.6	184.3 ± 46.7	0.000
HDL-C (mg/dL)	46.4 ± 9.0	34.1 ± 7.1	0.008
LDL-C (mg/dL)	106.1 ± 16.3	117.6 ± 37.9	0.000
HDL/LDL	0.45 ± 0.13	0.32 ± 0.14	0.006
Triglyceride (mg/dL)	102.6 ± 45.7	158.8 ± 88.7	0.025

Values are in mean ± SD.
